# Circulating free DNA as a diagnostic marker for echinococcosis: a systematic review and meta-analysis

**DOI:** 10.3389/fmicb.2024.1413532

**Published:** 2024-07-03

**Authors:** Xiaoqin Luo, Ping Jiang, Jideng Ma, Zian Li, Jianwu Zhou, Xiaoxing Wei, Jide A, Jinping Chai, Yanke Lv, Peng Cheng, Chunhua Cao, Xiangren A

**Affiliations:** ^1^Qinghai University, Xining, China; ^2^Department of Clinical Laboratory, Qinghai Provincial People’s Hospital, Xining, China

**Keywords:** echinococcosis, circulating free DNA, diagnostic marker, systematic review, meta-analysis

## Abstract

**Introduction:**

Echinococcosis is a chronic zoonotic disease caused by tapeworms of the genus *Echinococcus*. The World Health Organization (WHO) has identified encapsulated disease as one of 17 neglected diseases to be controlled or eliminated by 2050. There is no accurate, early, non-invasive molecular diagnostic method to detect echinococcosis. The feasibility of circulating free DNA as a diagnostic method for echinococcosis has yielded inconclusive results in a number of published studies. However, there has been no systematic evaluation to date assessing the overall performance of these assays. We report here the first meta-analysis assessing the diagnostic accuracy of cfDNA in plasma, serum, and urine for echinococcosis.

**Methods:**

We systematically searched PubMed, Embase, Cochrane Library, China National Knowledge Infrastructure (CNKI), and WeiPu databases up to 17 January 2024, for relevant studies. All analyses were performed using RevMan 5.3, Meta-DiSc 1.4, Stata 17.0, and R 4.3.1 software. The sensitivity, specificity, and other accuracy indicators of circulating free DNA for the diagnosis of echinococcosis were summarized. Subgroup analyses and meta-regression were performed to identify sources of heterogeneity.

**Results:**

A total of 7 studies included 218 patients with echinococcosis and 214 controls (156 healthy controls, 32 other disease controls (non-hydatid patients), and 26 non-study-targeted echinococcosis controls were included). Summary estimates of the diagnostic accuracy of cfDNA in the diagnosis of echinococcosis were as follows: sensitivity (SEN) of 0.51 (95% CI: 0.45–0.56); specificity (SPE) of 0.99 (95% CI: 0.97–0.99); positive likelihood ratio (PLR) of 11.82 (95% CI: 6.74–20.74); negative likelihood ratio (NLR) of 0.57 (95% CI: 0.41–0.80); diagnostic ratio (DOR) of 36.63 (95% CI: 13.75–97.59); and area under the curve (AUC) value of 0.98 (95% CI: 0.96–1.00).

**Conclusion:**

Existing evidence indicates that the combined specificity of circulating cfDNA for echinococcosis is high. However, the combined sensitivity performance is unsatisfactory due to significant inter-study heterogeneity. To strengthen the validity and accuracy of our findings, further large-scale prospective studies are required.

Systematic review registrationThe systematic review was registered in the International Prospective Register of Systematic Reviews PROSPERO [CRD42023454158]. https://www.crd.york.ac.uk/PROSPERO/.

## 1 Introduction

Echinococcosis is a chronic zoonotic infection that poses a serious public health problem, affecting many people around the world. There are two main types of worldwide transmission of the disease, cystic echinococcosis (CE) caused by *Echinococcus granulosus* transmitted by dogs and alveolar echinococcosis (AE) caused by *Echinococcus multiloculari* transmitted by foxes (Torgerson and Budke, 2003). Humans are incidental intermediate hosts in the life cycle of *Echinococcus*. CE, also known as cysticercosis, is the most common form worldwide. The latest reports indicate that the average annual incidence of CE from 1997 to 2020 was 0.64 cases per 100,000 people in Europe and 0.50 cases per 100,000 people in EU member states (Casulli et al., 2023). According to the United States classification developed by the World Health Organization Informal Working Group on Echinococcosis (WHOIWGE), CE encapsulations are classified into five types, CE1 through CE5 (Brunetti and Junghanss, 2009), which can cause varying degrees of signs and symptoms. In domestic animals, clinical signs are usually mild, and infection is usually detected during routine meat inspection (Abdulhameed et al., 2018). CE in humans is often considered a chronic disease, and most infected patients are asymptomatic, leading to an underestimation of the total number of infected individuals (Conraths et al., 2016; Borhani et al., 2021). AE is a vesicular coccidioidomycosis caused by *Enterobacteriaceae multiforme* and is spread primarily among wild carnivores (mainly red foxes). However, domestic dogs or small rodents may also be intermediate hosts (Romig et al., 2017; Wen et al., 2019). The form of AE is more complex and fatal as compared to CE and is classified into different PNM stages, denoting the extension of the parasitic mass in the liver (P), the involvement of neighboring organs (N), and metastases (M), which include parasitic lesions, neighboring organs, and metastases (Brunetti et al., 2010). Epidemiologic analyses indicate that AE is primarily distributed in the Northern Hemisphere and is a public health problem in Central and Eastern Europe, the Near East, Russia, China (especially the Tibetan Plateau), and northern Japan (Brunetti et al., 2010; Vuitton et al., 2016; Deplazes et al., 2017). It has been reported that 91% of the new cases of AE each year are from China (Conraths et al., 2016). AE exhibits aggressive growth and metastasis to other organs, leading to a mortality rate of up to 90% within 15 years in inadequately treated patients with AE, which has led to the disease being referred to as “worm cancer” (Torgerson et al., 2008; McManus et al., 2012; Bulakci et al., 2016; Yuan et al., 2020).

Currently, the diagnosis of encopresis is based on clinical manifestations, imaging, and immunodiagnostic tests (Brunetti and Junghanss, 2009; Brunetti et al., 2010). Imaging tests commonly used are ultrasonography, CT, and MRI, with ultrasonography being the most commonly used due to its low cost and rapid diagnostic advantages (Holcman and Heath, 1997; Casulli et al., 2019a; Wen et al., 2019). However, for the most part, only relatively large cysts are analyzed by imaging in patients with CE in the advanced stages of the disease, and it is difficult for this testing technique to differentiate between echinococcal tapeworm cysts and other types of cysts. In addition, several immunologic methods have been developed for the detection of anti-*Echinococcus* antibodies, which have led to the promise of serologic testing for earlier diagnosis than imaging techniques (Brunetti and Junghanss, 2009; Brunetti et al., 2010). However, the sensitivity and specificity of these immunologic assays vary under different conditions, especially in the case of CE (Gharbi et al., 1981; McManus et al., 2003; Polat et al., 2003; Cattaneo et al., 2013; Grüner et al., 2017; Brunetti et al., 2018; Stojkovic et al., 2018; Engler et al., 2019). In addition, preoperative pathologic examination of biopsy samples is not recommended due to the risk of propagation of proto-cephalic nodes and allergic reactions during the biopsy procedure (Raether and Hanel, 2003). This is coupled with the fact that the clinical manifestations of echinococcosis are not specific, and patients often develop symptoms only in the later stages of the disease. As a result, diagnosis of encapsulated disease is difficult, especially in the early stages of the disease (McManus et al., 2003), although for CE, the world’s most prevalent type of cysticercosis, the United States provides reliable information on the location, number, size, and stage of the cysts (Brunetti and Junghanss, 2009; Grüner et al., 2017; Stojkovic et al., 2018). As a result, the WHO has recognized echinococcosis as a largely neglected disease (Da Silva, 2010; Casulli et al., 2019a,b). Based on this, there is an urgent need to develop sensitive and specific diagnostic methods or biomarkers for the early detection of encapsulated diseases. In recent years, with the rapid advances in liquid biopsy analysis and analytical techniques, a number of studies on the detection of cfDNA, the circulating molecule of the parasite, in samples such as serum, plasma, and urine from patients have been reported, which promises to provide new avenues for non-invasive detection of echinococcosis.

cfDNA is an extracellular free DNA molecule present in body fluids such as blood, urine, and saliva. It is a detectable fragment of nucleic acid released from cells into the circulation. This release can occur passively due to various forms of cell death or through active secretion (Pajek et al., 2010; Wang et al., 2015; Aucamp et al., 2018; Otandault et al., 2019; Grabuschnig et al., 2020) cfDNA includes genomic (*cf*-gDNA) and mitochondrial DNA (*cf*-mtDNA), depending on the source (Bronkhorst et al., 2021). In recent years, cfDNA has been used as a marker in diagnostic studies in the areas of cancer (Jamshidi et al., 2022), prenatal screening (Bianchi and Chiu, 2018), infection, and injury (Andargie et al., 2021), and it also has great potential for clinical applications in tumor diagnosis, prognosis, and treatment monitoring (Wagner, 2012; Pietrasz et al., 2017; Corcoran and Chabner, 2018; Mann et al., 2018; Valpione et al., 2018; Osumi et al., 2019). In addition, cfDNA has been reported to be detected in a variety of parasitic diseases, such as Plasmodium, Trypanosoma, and *Echinococcus granulosus,* and is considered a diagnostic tool for human parasitic infections (Gal et al., 2001; Chaya and Parija, 2014; Ngotho et al., 2015; Weerakoon and McManus, 2016). However, the feasibility of cfDNA as a diagnostic method for echinococcosis has been inconsistent in the results of a number of published studies, and no previous meta-analysis in the literature has covered this research question. In this study, we utilized data from multiple studies in a meta-analysis to systematically assess the potential of using circulating cfDNA as a non-invasive biomarker for the diagnosis of echinococcosis.

## 2 Methods

This review adhered to the Preferred Reporting Items for Systematic Reviews and Meta-Analyses (PRISMA) criteria (Moher et al., 2009). The proposed methodology for the systematic review was registered in the International Prospective Register of Systematic Reviews, PROSPERO [CRD42023454158].

### 2.1 Literature search

A combination of MeSH terms and entry terms was used to search mainstream databases, including PubMed, EMBASE, and the Cochrane Library. We also searched Chinese databases, including the China National Knowledge Infrastructure (CNKI) and WeiPu databases, without language limitation. No limitation was set on the start date for the publications, and the search ended on 17 January 2024. The following retrieval indexes were used: (echinococcosis OR Echinococcoses OR *Echinococcus* Infection OR Cystic Echinococcosis OR Hydatidosis OR Hydatid Cyst OR Hydatid Disease OR *Echinococcus* Granulosus Infection OR Cystic Echinococcoses OR Hydatidoses OR alveolar echinococcosis OR Hepatic Echinococcosis OR Hepatic Hydatidosis OR Hepatic Hydatid Cyst OR Hepatic Alveolar Echinococcosis) AND (cfDNA OR cirDNA OR Cell Free DNA OR Circulating Nucleic Acid OR Cell Free Nucleic Acid OR Circulating Cell Free Nucleic Acid OR Circulating Cell-free DNA OR Cell Free Deoxyribonucleic Acid OR Circulating DNA). In addition, reference lists of the included articles and potentially eligible studies based on the identified review articles were cross-checked to search for additional relevant studies that were not detected by the original literature search.

### 2.2 Inclusion and exclusion criteria

The following inclusion criteria were used in this meta-analysis: (Torgerson and Budke, 2003) studies that evaluated the diagnostic accuracy of quantitative analysis of cfDNA for *Echinococcus* infection; (Casulli et al., 2023) studies that reported sensitivity and specificity or from which these metrics could be calculated from 2 × 2 contingency tables; (Brunetti and Junghanss, 2009) studies that provided absolute numbers of true-positive (TP), false-positive (FP), true-negative (TN), and false-negative (FN) cases were provided; (Abdulhameed et al., 2018) studies from which full dataset could be retrieved from the publication and the full-text article was available; (Conraths et al., 2016) only studies that included at least eight echinococcosis patients were selected, as very small sample size may lead to selection bias. Exclusion criteria included: (Torgerson and Budke, 2003) studies with incomplete data, data that could not be retrieved or reconstructed for 2 × 2 tables; (Casulli et al., 2023) studies that overlapped the included studies (i.e., studies from the same institution, study group, and with the same results); (Brunetti and Junghanss, 2009) unsuitable publication types, including letters, comments, editorials, and expert opinions; reviews without original data; case reports or studies with fewer than eight patients.

### 2.3 Data retrieval

Two reviewers (XQ Luo and P Jiang) independently retrieved data from all eligible studies. First, duplicated publications were removed by manual searching, and then we checked again to ensure that there were no duplicate records. The remaining articles were evaluated based on their titles and abstracts and were included for full-text assessment if they met all eligibility criteria based on the PICOS principle: (1) Participants: patients with echinococcosis; (2) Interventions: the detection of cfDNA; (3) Comparisons: non-echinococcosis controls; (4) Outcomes: diagnostic sensitivity (SEN) and specificity (SPE), or the number of true-positive (TP), false-positive (FP), true-negative (TN), and false-negative (FN) results of the diagnostic test; and (5) Study design: diagnostic research. Any article was excluded during the full-text assessment if the data were found to be insufficient. In addition, we conducted a manual search for potentially eligible studies based on the identified review articles’ reference lists.

### 2.4 Quality assessment

To assess the methodological quality of each study and the potential risk of bias, we used the Quality Assessment of Diagnostic Accuracy Studies-2 (QUADAS-2) tool (Moher et al., 2009; Whiting et al., 2011), which has been widely used since its publication in 2011 and has been integrated into the Cochrane Collaboration dedicated software RevMan 5.2 in 2012 (Yang et al., 2001; Whiting et al., 2011; Zhu et al., 2012). The QUADAS-2 tool is comprised of four key domains: patient selection, index test, reference standard, and flow and timing. We used seven items from the QUADAS-2 to evaluate the quality of the included studies. Quality assessment was undertaken by one reviewer and checked by a second reviewer, with disagreements resolved by a third reviewer. The process of quality assessment and mapping was performed with RevMan 5.3 software.

### 2.5 Data extraction and statistical analysis

The process of data extraction was independently completed by two researchers, with one extracting the data and another rechecking the data. Statistical analysis was performed utilizing Meta-DiSc 1.4 (Cochrane Colloquium, Barcelona, Spain), Stata 17.0 (Stata Corporation, College Station, United States), and R 4.3.1 (R Development Core Team University of Akron, New Zealand) software (RRID:SCR_001905). The original data were extracted with a standardized form (Midgette et al., 1993; Irwig et al., 1994), which included the following items: (1) basic characteristics of studies, including last name of the first author, year of publication, country of origin, methods of detection, type of echinococcosis (AE/CE), type of specimens (plasma/serum/urine), specificity, sensitivity, TP, FP, TN, and FN; (2) diagnostic performance: The bivariate meta-analysis model was employed to summarize the sensitivity (SEN), specificity (SPN), diagnostic odds ratio (DOR), positive likelihood ratio (PLR), and negative likelihood ratio (NLR). Meanwhile, the bivariate SROC and its 95% confidence interval (95% CI) were generated by plotting the sensitivity and specificity of each of the included studies (Arends et al., 2008). The area under the curve (AUC) was used for grading the overall accuracy as a potential summary of the SROC curve. In addition, the threshold effect was detected by Spearman’s correlation coefficient, and a *p*-value of less than 0.05 indicated a significant threshold effect. Heterogeneity between studies was assessed using the chi-square test and I^2^ test. *p*-values less than 0.1 or *I*^2^ values greater than 50% indicated significant heterogeneity. Studies with I^2^ values of 0–25%, 26–50%, 51–75%, and 75–100% indicated no, low, moderate, and substantial heterogeneity, respectively (Midgette et al., 1993), and subgroup analyses and regression analyses were then performed according to the expertise to determine the heterogeneity sources of heterogeneity. Funnel plots and Egger’s linear regression were used to evaluate the presence of publication bias in the included studies. Sensitivity analyses were performed by reducing one document at a time to evaluate the stability of this analysis. All statistical tests were two-sided, and *p* < 0.05 was considered statistically significant (Higgins et al., 2003; Deeks et al., 2005; Reitsma et al., 2005; Zamora et al., 2006).

## 3 Results

### 3.1 Search results

**Figure 1:**
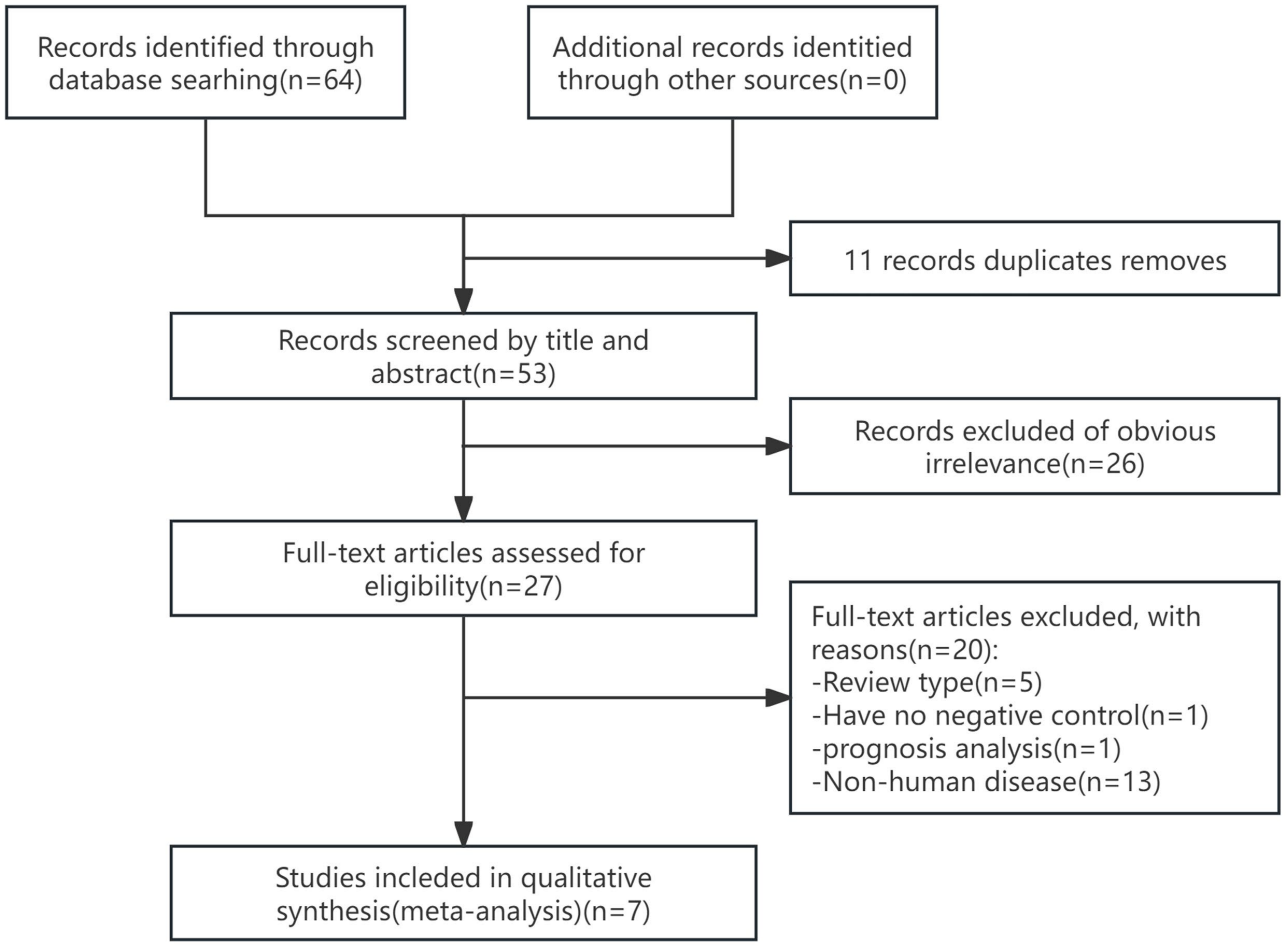
PRISMA flowchart for screening studies of Echinococcosis.

After the initial search retrieved a total of 64 publications by MeSH items and titles, 64 articles were selected (PubMed 44, Embase 12, Cochrane 0, CNKI 5, CQVIP 3), and after the removal of duplicates, 11 repetitive articles were excluded. The abstracts, keywords, and full texts of the remaining 53 studies were subsequently reviewed through to exclusion of 47 studies (five were review-type, one had no negative controls, one for prognostic analysis, and the others were non-diagnostic or non-human disease). All reviews and references from the remaining articles that did not meet the exclusion criteria were then cross-checked to determine that there were no additional articles that met the inclusion criteria. We ended up with seven studies. The flowchart for literature search and study selection is shown in Figure 1.

### 3.2 Characteristics of the included studies and quality assessments

In this meta-analysis, we ultimately included seven diagnostic studies (Chaya and Parija, 2014; Baraquin et al., 2018; Toribio et al., 2020; Wan et al., 2020; Wang et al., 2020; Fan et al., 2021; Li et al., 2022), a total of 218 hydatid patients and 156 healthy controls, 32 other disease controls (non-hydatid patients), and 26 non-study-targeted echinococcosis controls. The 218 echinococcosis patients were composed of 154 alveolar echinococcosis patients and 64 cystic echinococcosis patients. According to the diagnostic criteria recommended in the expert consensus (Baraquin et al., 2018), the diagnosis of all patients with echinococcosis was based on one or more examination methods of histopathology, imaging, and serology (one study (Wang et al., 2020) was confirmed by e-mail contact with the authors themselves). Regarding the origin of the studies, four were conducted in China (northwest; Wan et al., 2020; Wang et al., 2020; Fan et al., 2021; Li et al., 2022), one in France (Baraquin et al., 2018), one in India (Chaya and Parija, 2014) and another one in Peru (Toribio et al., 2020). All studies were published between 2014 and 2022, and the number of echinococcosis patients in each study ranged from 8 to 105. Methods of cfDNA isolation and detection also varied among the included studies. Commercial kits were used in all but one study (Chaya and Parija, 2014). In addition, two used PCR (Chaya and Parija, 2014; Toribio et al., 2020), two used qPCR to measure circulating cfDNA (Baraquin et al., 2018; Wang et al., 2020), one study added the DDPCR detection method (Baraquin et al., 2018), one used multiple PCR followed by NGS detection (Wan et al., 2020), one used one-tube nested MGB Probe real-time PCR (Li et al., 2022), and another one used the NGS/sequence method to measure circulating cfDNA (Fan et al., 2021). In terms of diagnostic accuracy of cfDNA in echinococcosis, three studies used plasma cfDNA (Wan et al., 2020; Fan et al., 2021; Li et al., 2022), two studies used serum cfDNA (Baraquin et al., 2018; Wang et al., 2020), and another one used urinary cfDNA (Toribio et al., 2020); the remaining study used both urinary and serum cfDNA (Chaya and Parija, 2014).

**Figure 2:**
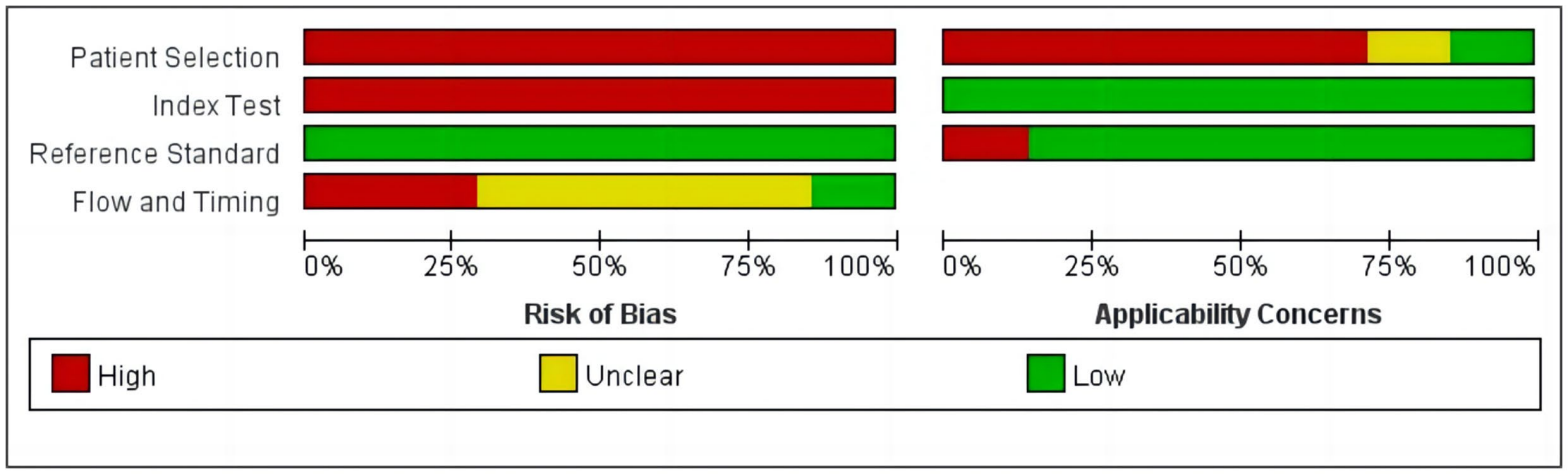
Quality assessment of the studies selected for the meta-analysis (QUADAS-2).

On the selection of target sequences, one used the *Echinococcus* mitochondrial DNA sequence NAD1 (Chaya and Parija, 2014), and one used both the mitochondrial DNA sequence NAD5 and the nuclear sequence snRNA specific to *Echinococcus* (Baraquin et al., 2018). One does not screen for specific hydatid sequences (Fan et al., 2021); instead, all possible unique sequences of *Echinococcus* were preserved. Specific sequences of *Echinococcus* were screened, respectively, from the remaining four studies. For data collection, all studies had a retrospective design. Most studies did not report how they collected the data or whether they were blinded. The main characteristics of the included studies are shown in Table 1. We found that, overall, the quality assessment results of most of the studies had a moderate–high quality using the Quadas-2 tool. There was a high risk of bias in the domain of “Patient Selection.” According to the Quadas-2 team statement, the ideal diagnostic study should recruit a proportion of suspected patients (“Hard-to-diagnose patients”) to reduce the risk of bias (Whiting et al., 2011). However, all of our studies included patients with a confirmed diagnosis, and these included studies were not described as performing blinding, thus resulting in a high risk of bias in both areas. In addition, the risk of bias in the “Process and Scheduling” domain was unclear in most of the studies because the time intervals were not described. The risk of bias was low in all of the “Reference Testing” domains. In the applicability domain, “Sample selection” showed a high level of concern, mainly considering that the samples of all articles were from relatively restricted areas and may not be representative of performance in all high-prevalence areas. The remaining domains of applicability all showed a low level of concern. The results of the quality assessment are shown in Figure 2.

**Table 1:** Included study characteristics.

Study	Year	EG/EM	Clinical sample types	Nationality	Original diagnostic technique	Size of participants	CfDNA isolation kits	Methods used	Target sequence	TN	FP	FN	TP	Sensitivity (%)	Specificity (%)
Runle Li	2022	EM	Plasma	China	Based on imaging and Immunological diagnosis	13 AE + 10 CE patients, 30 healthy people	NucleoSnap DNA Plasma Kit (Macherey-Nagel, Germany)	One-tube nested MGB probe real-time PCR	CBLO020001206.1	40	0	2	11	84.6	100
Haining Fan	2020	EM	Plasma	China	Diagnosis based on pathology	105 AE + 16 CE patients, 4 liver cancer, 4 gallstones, and 20 healthy volunteers	QIAamp MinElute ccfDNA Mini Kit	DNA sequencing	Em-unique reads	40	4	0	105	100	90.9
Zhengqing Wan	2020	EG	Plasma	China	Diagnosed by imaging, immunology, and pathology	24 patients (19 CE/5 AE), 27 healthy +9 Schistosoma controls	QIAGEN circulating nucleic acid kit	Targeted multiplex PCR + NGS	70–100 bp of repeat regions (e.g., >CL11Contig2)	36	0	4	15	78.9	100
		EM								36	0	1	4	80	100
		EG						NGS only	uniquely mapped to EG/EM database	11	0	10	1	9.1	100
		EM								11	0	1	4	80	100
Luz Toribio	2020	EG	Urine	Peru	Imaging-based diagnosis	12 CE patients +25 controls	QIamp Mini Kit	PCR	133 bp fragment of the EG EgG1 Hae III repeat region	24	1	3	9	75	96
Wang Ying	2020	EG	Serum	China	Based on imaging and surgical confirmation	8 CE patients +8 controls	QIAamp Circulating Nucleic Acid Kit	qPCR	101 bp (EG sequence)	7	1	3	5	62.5	87.5
Alice Baraquin	2018	EM	Serum	France	Based on imaging and Immunological diagnosis	31 AE patients +36 controls	QIAamp Circulating Nucleic Acid Kit	qPCR	U1 small nuclear (sn)RNA (nuclear)	36	0	26	5	16.1	100
								droplet digital PCR (ddPCR)	U1 small nuclear (sn)RNA (nuclear)	36	0	24	7	22.6	100
								qPCR	nad5 (mitochondrial)	36	0	28	3	9.7	100
								droplet digital PCR (ddPCR)	nad5 (mitochondrial)	36	0	27	4	12.9	100
DR Chaya	2014	EG	Urine	India	Based on imaging and surgical confirmation	25 CE patients, 10 controls +15 Other parasites controls	Phenol–chloroform–isoamyl alcohol	PCR	450 bp nad1 (mitochondrial)	25	0	25	0	20	100
			Serum							25	0	20	5	2	100

EG, *E. granulosus*; EM, *E. multilocularis*; TP, true-positive; FP, false-positive; FN, false-negative; TN, true-negative.

### 3.3 Tests for heterogeneity

When DOR was used as the effect size to analyze the heterogeneity of echinococcosis, the Q-test showed a Cochran-Q of 19.61, *p* = 0.11, while the I^2^ was 33.7%, suggesting that inter-study heterogeneity at this point was low. When the sensitivity effect value was used as the evaluation index, its chi-square test *p*-value was 0.00, and I^2^ was 95.4%, suggesting a high degree of heterogeneity in terms of sensitivity among the studies. Therefore, we chose a random-effects model for the following analysis and explored the sources of heterogeneity through subgroup and regression analyses.

### 3.4 Diagnostic accuracy

**Figure 3:**
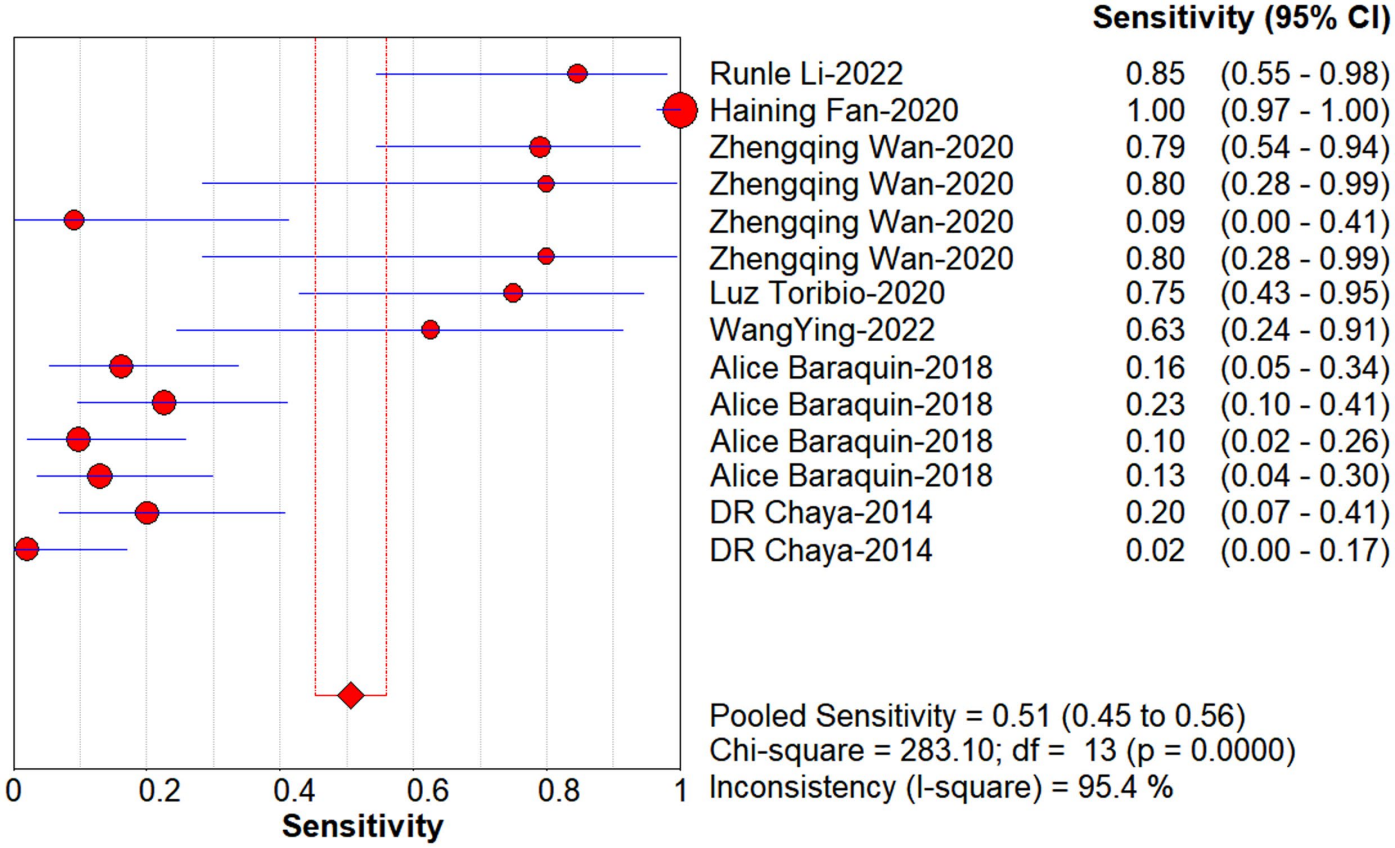
Forest plot of sensitivity for quantitative analysis of circulating cell-free DNA in the diagnosis of Echinococcosis.

**Figure 4:**
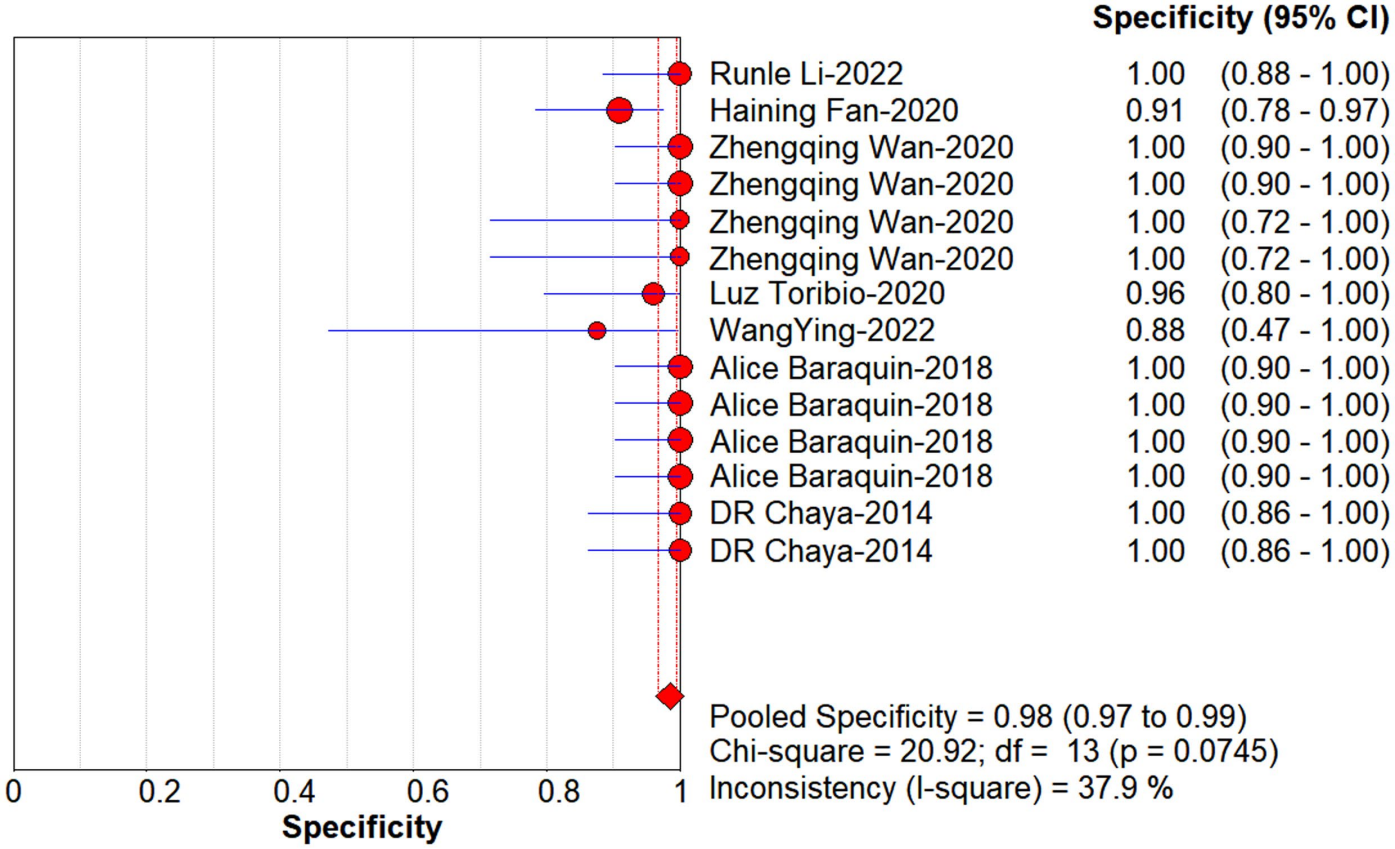
Forest plot of specificity for quantitative analysis of circulating cell-free DNA in the diagnosis of Echinococcosis.

**Figure 5:**
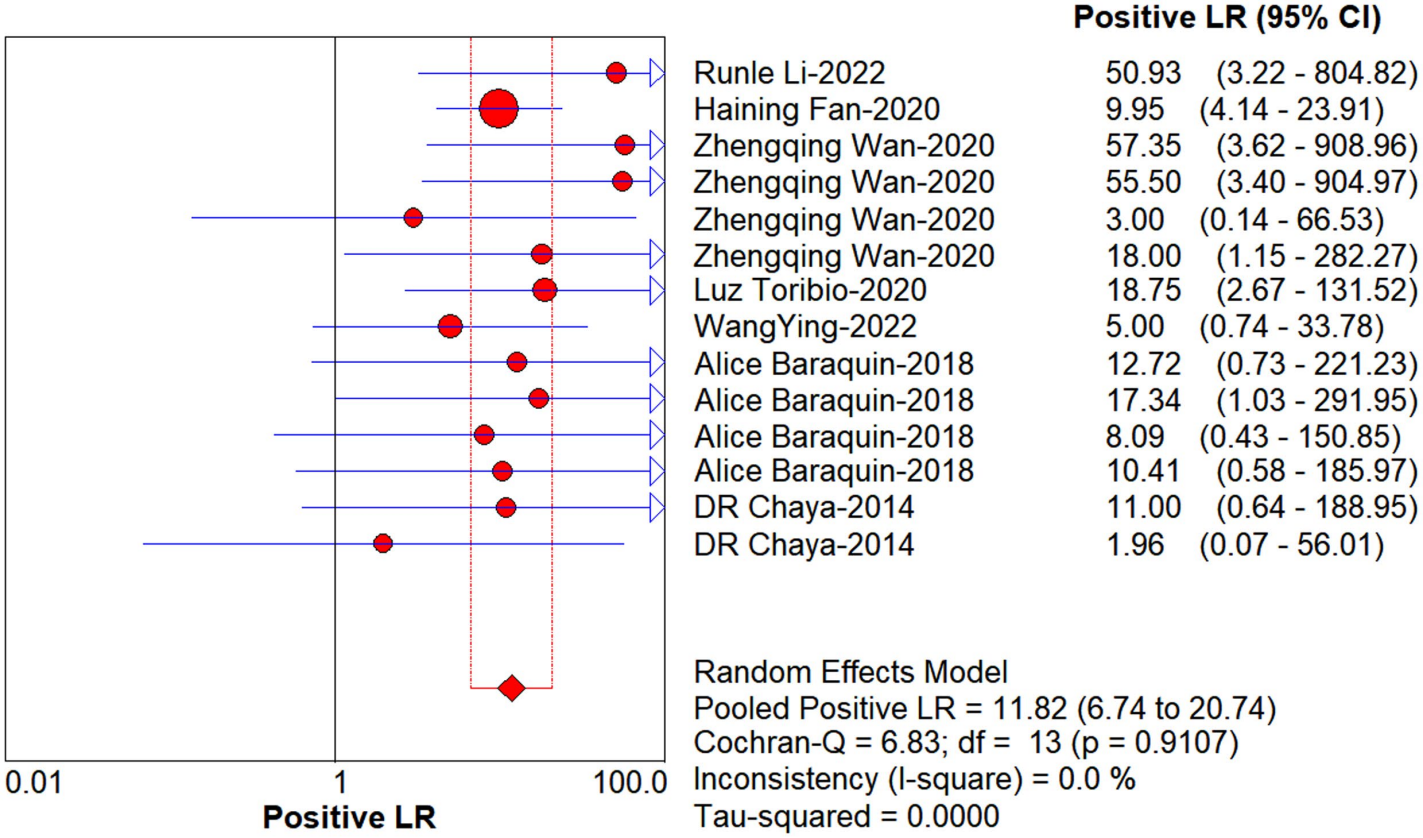
Forest plot of estimate PLR for quantitative analysis of circulating cell-free DNA in the diagnosis of Echinococcosis.

**Figure 6:**
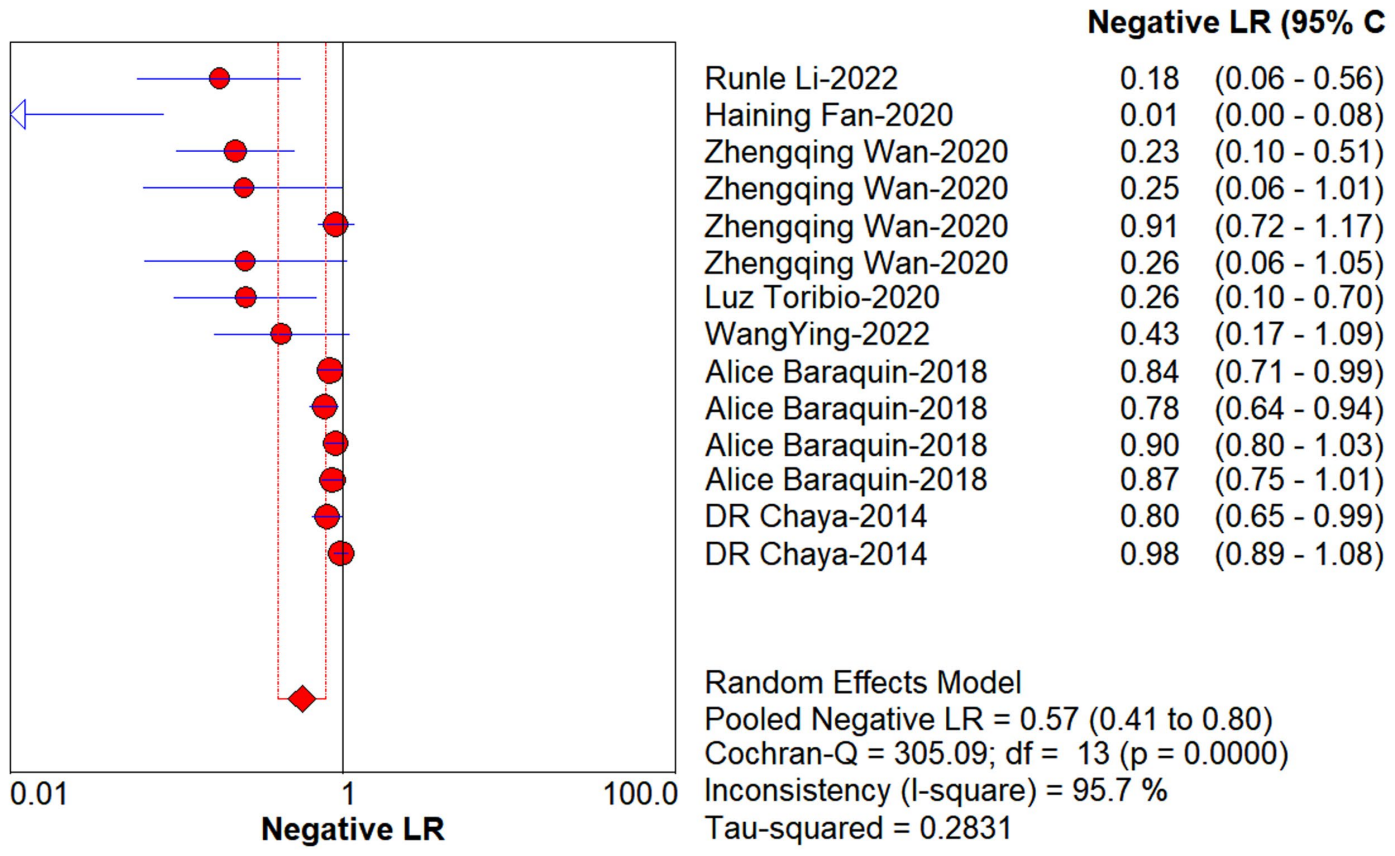
Forest plot of estimate NLR for quantitative analysis of circulating cell-free DNA in the diagnosis of Echinococcosis.

**Figure 7:**
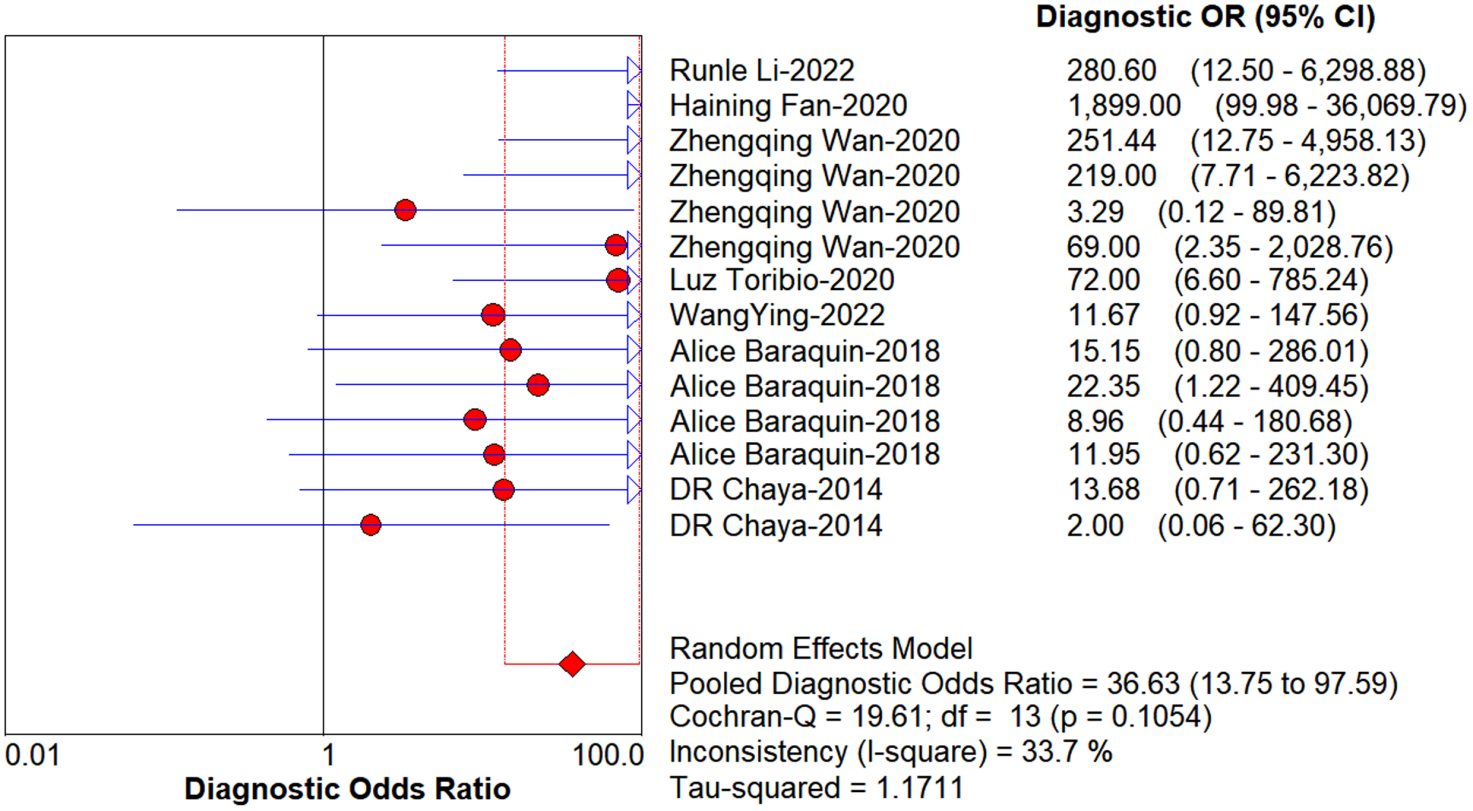
Forest plot of DOR for quantitative analysis of circulating cell-free DNA in the diagnosis of Echinococcosis.

**Figure 8:**
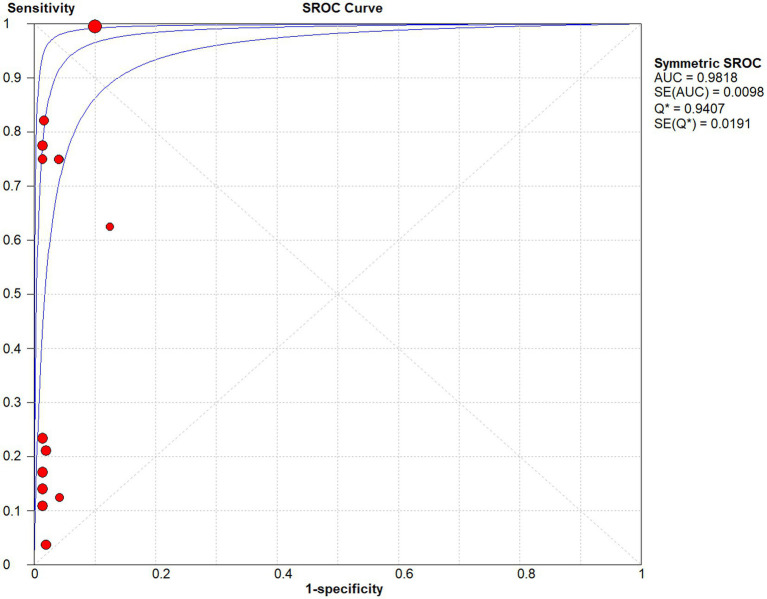
The SROC curve for quantitative analysis of circulating cell-free DNA in the diagnosis of Echinococcosis.

Studies as per the inclusion criteria were subjected to the analysis, regardless of their subtype and sample sources. We derive the pooled sensitivity and specificity as 0.51 (95% CI: 0.45–0.56; Figure 3) and 0.99 (95% CI: 0.97–0.99; Figure 4), respectively. The pooled PLR was 11.82 (95% CI: 6.74–20.74; Figure 5), NLR was 0.57 (95% CI: 0.41–0.80; Figure 6), and diagnostic odds ratio was 36.63 (95% CI: 13.75–97.59; Figure 7). In addition, the SROC curve for the included studies is shown in Figure 8. The AUC value was 0.98 (95% CI: 0.96–1.0), indicating relatively high accuracy of quantitative analysis of circulating cfDNA for echinococcosis diagnosis.

### 3.5 Analysis of the sources of inter-study heterogeneity

#### 3.5.1 Existence of threshold effect

As shown in the SROC curves of echinococcosis, the corresponding points of each study are scattered and do not have a “shoulder-arm” appearance. Spearman’s correlation coefficients *P* between the logarithm of sensitivity and the logarithm of (1-specificity) were calculated, and *p* = 0.462, confirming that the threshold effect was not significant and the heterogeneity was caused by other reasons.

#### 3.5.2 Subgroup analyses

Subgroup analyses were performed for different subtypes, which included country origin (China or other countries), sample type (plasma, serum, or urine), disease type (AE or CE), year of publication (pre-2020 or post-2020), and sample size (N ≤ 50 or N > 50). We found that the overall accuracy was better in the Chinese population compared to other national populations, with sensitivities of 0.87 versus 0.18, specificities of 0.97 versus 0.99, PLRs of 12.09 versus 11.23, NLRs of 0.21 versus 0.85, DORs of 95.77 versus 16.10, and AUC values of 0.96 versus 0.87, respectively, and we also found that compared to the plasma-based assays showed a higher level of accuracy compared to serum- and urine-based assays, with sensitivities of 0.87, 0.19, and 0.25, PLRs of 13.71, 8.93, and 9.29, NLRs of 0.17, 0.85, and 0.51, and DORs of 149.03, 13.36, and 14.96, with AUC values of 0.97, 0.86, and 0.72, respectively. These results indicate that the best source of cfDNA detection for echinococcosis was plasma. However, there was no significant difference in specificities (0.98, 0.99, and 0.98). In addition, subgroup analysis based on disease type showed that cfDNA testing was more accurate for diagnosis in the AE group, with sensitivities of 0.57 versus 0.35, PLRs of 13.07 versus 9.47, NLRs of 0.54 versus 0.57, DORs of 62.12 versus 19.38, and AUC values of 0.95 versus 0.90, respectively, and again with no significant difference in specificity (0.99 and 0.99). We even found that subgroup analyses based on year of publication and sample size showed that studies done after 2020 were more accurate, with sensitivities of 0.87 versus 0.14, specificities of 0.97 and 1, PLRs of 12.67 versus 9.25, NLRs of 0.22 versus 0.88, and DORs of 91.20 versus 10.81, respectively, and AUC values of 0.96 versus 0.84; whereas the larger sample size group was more accurate in the diagnosis of echinococcosis compared to the smaller sample size group, with sensitivities of 0.58 versus 0.31, specificities of 0.98 versus 0.99, PLRs of 12.86 versus 10.13, NLRs of 0.53 versus 0.58, DORs of 64.28 versus 21.27, and AUC values of 0.95 versus 0.88. Therefore, the results of the above analysis should be referred to with caution. The summary data of sensitivity, specificity, PLR, NLR, DOR, and AUC for each subgroup are shown in Table 2.

**Table 2:** Subgroup analyses performed to identify potential sources of heterogeneity.

Variables	No. of dataset	SEN (95% CI)	SPE (95% CI)	PLR (95% CI)	NLR (95% CI)	DOR (95% CI)	AUC
Overall	14	0.51 (0.45–0.56)	0.99 (0.97–0.99)	11.82 (6.74–20.74)	0.57 (0.41–0.80)	36.63 (13.75–97.59)	0.98
Country origin							
China	7	0.87 (0.81–0.92)	0.97 (0.94–0.991)	12.09 (6.16–23.73)	0.21 (0.04–1.14)	95.77 (18.93–484.42)	0.96
Non-China	7	0.18 (0.13–0.24)	1.00 (0.98–1.00)	11.22 (4.05–31.12)	0.85 (0.75–0.97)	16.10 (5.39–48.16)	0.87
Sample type							
Plasma	6	0.89 (0.83–0.93)	0.98 (0.94–0.99)	13.71 (6.67–28.17)	0.17 (0.02–1.51)	149.03 (27.55–806.31)	0.97
Serum	6	0.19 (0.13–0.25)	0.99 (0.97–1.00)	8.93 (3.08–25.90)	0.85 (0.79–0.91)	13.36 (4.14–43.11)	0.86
Urine	2	0.25 (0.13–0.42)	0.98 (0.89–1.00)	9.29 (1.19–72.43)	0.51 (0.02–17.02)	14.96 (0.45–493.97)	0.72
Disease type							
AE	8	0.57 (0.50–0.63)	0.99 (0.96–1.00)	13.07 (6.64–25.74)	0.54 (0.33–0.87)	62.12 (16.19–238.38)	0.95
CE	6	0.35 (0.26–0.46)	0.99 (0.95–1.00)	9.47 (3.46–25.92)	0.57 (0.33–1.01)	19.38 (4.81–78.14)	0.90
Year of publication							
Pre-2020	6	0.14 (0.09–0.20)	1.00 (0.98–1.00)	9.25 (2.79–30.61)	0.88 (0.81–0.96)	10.81 (3.15–37.08)	0.84
Post-2020	8	0.87 (0.81–0.91)	0.97 (0.94–0.99)	12.67 (6.70–23.96)	0.22 (0.05–0.91)	91.20 (23.43–354.98)	0.96
Sample size							
N ≤ 50	7	0.31 (0.22–0.42)	0.99 (0.95–1.00)	10.13 (3.93–26.13)	0.58 (0.36–0.96)	21.27 (6.46–70.01)	0.88
N > 50	7	0.58 (0.51–0.64)	0.98 (0.96–1.00)	12.86 (6.39–25.86)	0.53 (0.32–0.88)	64.28 (13.93–296.66)	0.95

SEN, sensitivity; SPE, specificity; PLR, positive likelihood ratio; NLR, negative likelihood ratio; DOR, diagnostic odds ratio; AUC, area under the curve.

#### 3.5.3 Meta-regression analysis for heterogeneity

To explore possible sources of heterogeneity across these seven studies, we further assessed the impacts of the following specific variables on heterogeneity using meta-regression analyses: “country origin,” “sample type,” “disease type,” “year of publication,” “sample size,” “assay methods” and “target sequence.” The results showed that there were statistically significant differences in the heterogeneity of the studies based on “country origin,” “sample type,” “year of publication,” “assay methods,” and “target sequence.” The remaining variables did not lead to statistically significant effects between studies (Table 3). In addition, the differences in gender, age, ethnicity, echinococcosis stage, metastasis, and specific cfDNA detection methods of the echinococcosis patients included in the studies could not be further analyzed because most of the papers did not provide complete data or had unidentifiable details.

**Table 3:** Meta-regression performed to identify potential sources of heterogeneity.

Covariates	Estimate	Std. Error	*Z*	*P*	95% CI
Whether domestic	−2.44	0.77	−3.18	0.00	−3.94 to 0.94
Publication year	−2.65	0.49	−5.47	0.00	−3.60 to 1.70
Sample type	−1.50	0.71	−2.12	0.03	−2.89 to 0.11
Method	0.75	0.23	3.26	0.00	0.30 to 1.20
Sequence	1.22	0.28	4.34	0.00	0.67 to 1.77
Volume	−0.42	1.13	−0.37	0.71	−2.63 to 1.79
Disease type	−0.65	1.14	−0.57	0.57	−2.89 to 1.59

### 3.6 Sensitivity analysis

Sensitivity analyses were performed by reducing one paper at a time to assess the impact of individual studies on the meta-analysis. Table 4 shows the combined DOR and its 95% CI calculated after removing one article, which shows that the combined DOR did not change significantly regardless of which article was removed, suggesting that the results of the present analysis did not depend too much on a particular study, and the conclusion was stable.

**Table 4:** The influence of each study on the outcome of the meta-analysis.

Study omitted	DOR	95% CI
Haining Fan (2020)	31.83	13.62–74.39
Runle Li (2022)	38.02	14.04–102.99
Zhengqing Wan (2020)	37.99	13.96–103.40
Zhengqing Wan (2020)	39.3	14.34–107.71
Zhengqing Wan (2020)	52.31	20.08–136.24
Zhengqing Wan (2020)	42.58	15.12–119.95
Luz Toribio (2020)	41.58	14.35–120.45
Wang Ying (2020)	50.39	18.07–140.52
Alice Baraquin (2018)	48.02	17.11–134.78
Alice Baraquin (2018)	46.53	16.39–132.07
Alice Baraquin (2018)	49.92	18.18–137.10
Alice Baraquin (2018)	48.9	17.58–136.04
DR Chaya (2014)	48.39	17.30–135.32
DR Chaya (2014)	43.86	16.72–115.04
Combined	43.86	16.72–115.04

### 3.7 Publication bias estimate

**Figure 9:**
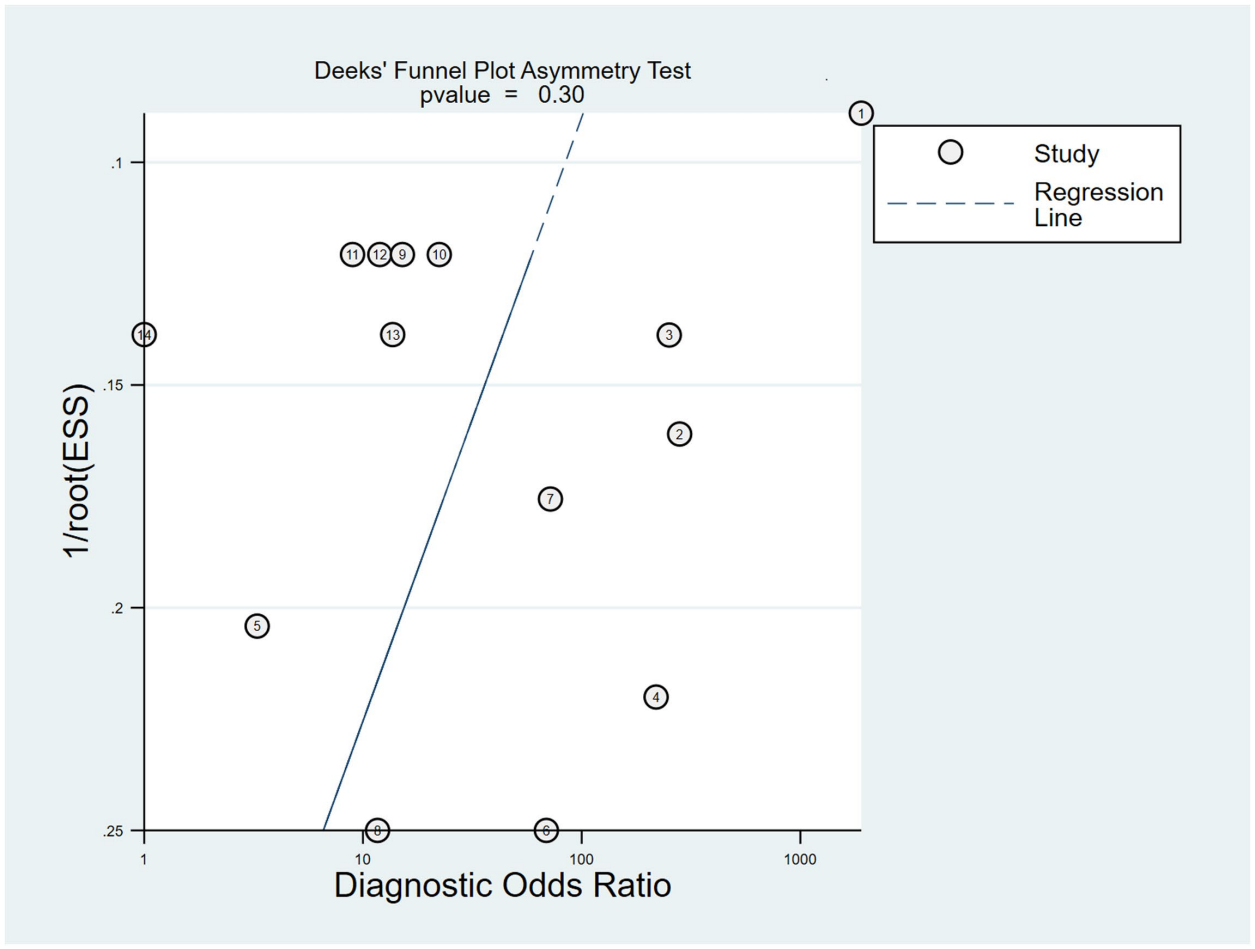
The Deeks’ funnel plot for the assessment of potential publication bias of the included studies.

**Figure 10:**
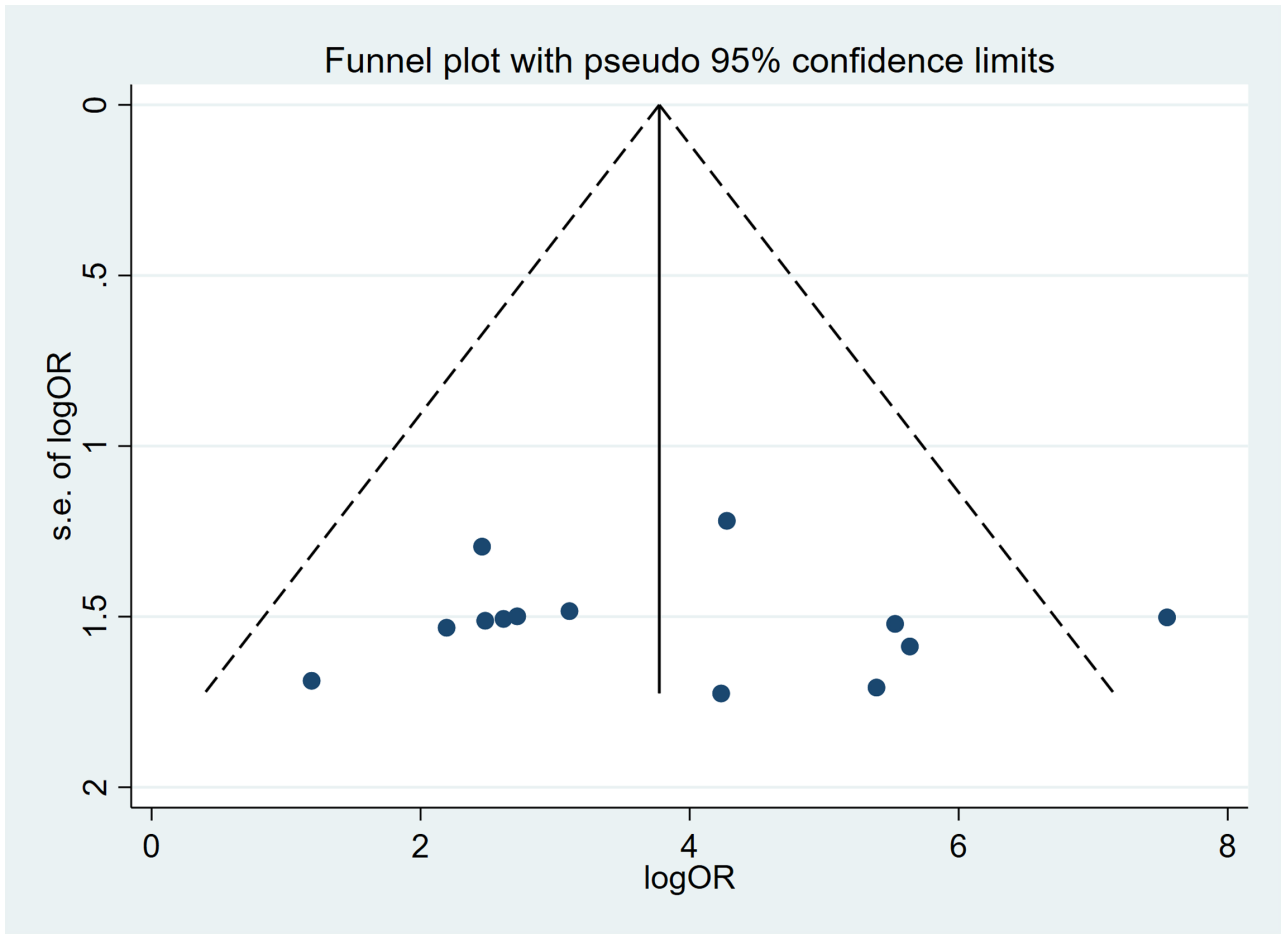
Funnel plot for the analysis of the level of bias in meta-analytical data for cfDNA in Echinococcosis.

Publication bias is considered a parameter that affects diagnostic performance. We assessed publication bias by using a scatterplot of the inverse of the square root of the effective sample size (1/ESS1/2) versus the diagnostic log ratio (lnDOR). When there is no publication bias, it should have a symmetrical funnel shape (Deeks et al., 2005). All analyses were performed using Stata 17.0 for Windows and R software, including the user-written commands midas, metabias, metafunnel, metaninf, and the R-mada program package. In the present meta-analysis, we concluded that Deeks’ funnel plots did not provide evidence of publication bias (*p* = 0.30), suggesting that the likelihood of publication bias in the present meta-analysis was low (Figure 9). We also used the Egger test (*p* = 0.816), and no evidence of publication bias was observed. In addition, we found that a funnel plot using the standard error of the logarithm of the DOR (SELogDOR) as the horizontal coordinate and Log (DOR) as the vertical coordinate, as shown in Figure 10, showed that no publication bias was found either.

## 4 Discussion

Our study is the first meta-analysis and systematic assessment of the feasibility of cfDNA as a diagnostic tool for echinococcosis, although several reviews have described some aspects (Zhao et al., 2021; Hadipour et al., 2023). After careful screening of the 64 articles from the initial search, we ended up with seven articles on the diagnostic accuracy of cfDNA for echinococcosis. Among these studies Wan et al. (2020) studied two disease types, EM and EG, and methodologically used both NGS-only and NGS-combined multiplex PCR assays. Baraquin et al. (2018) studied two target sequences, nuclear/snRNA and mitochondrial DNA, and used two different assays, qPCR and ddPCR assays. Chaya and Parija (2014) studied and analyzed two specimen types: urine and serum. All studies had more complete information in terms of “country origin,” “sample type,” “disease type,” “year of publication,” “sample size,” “assay methods,” and “target sequence.” In addition, the differences in gender, age, ethnicity, echinococcosis stage, metastasis, and specific cfDNA detection methods of the echinococcosis patients included in the studies could not be further analyzed because most of the papers did not provide complete data or had unidentifiable details. After quality assessment using the QUADAS-2 tool, we found that there was a high risk of bias in the areas of “Patient Selection” and “Index Test.” In accordance with the recommendations of the QUADAS-2 assessment, our study did not include consecutive or randomized eligible patients with suspected disease to reduce the risk of bias, and therefore the risk of bias in this area is high (Wu et al., 2013). In addition, there is a higher risk of bias in the “index field” because the included studies did not clearly describe whether they were blinded. A relatively low risk of bias was observed in the areas of “Reference Standard” and “Flow and Timing.” In the area of applicability, “Sample Selection” shows a high level of concern, considering that the most recent studies are mostly from northwestern China and may not be representative of the whole. Then, we performed a meta-analysis of the included articles to evaluate the diagnostic efficacy of cfDNA for echinococcosis by combining the diagnostic effect sizes and fitting SROC curves. Factors that might influence the results of the studies were found by analyzing the inter-study heterogeneity and its sources. Finally, the credibility of this meta-analysis was assessed by sensitivity analysis and the detection of publication bias. Statistically, the pooled sensitivity and specificity of the circulating cfDNA assay were 0.51 (95% CI: 0.45–0.56) and 0.99 (95% CI: 0.97–0.99), respectively, suggesting that quantitative analysis of cfDNA has poor sensitivity but high specificity for the diagnosis of echinococcosis. Likelihood ratios (LRs) are indicators of the true nature of sensitivity and specificity; in most cases, likelihood ratios higher than 10 and lower than 0.1 are considered to provide strong evidence to determine or exclude a diagnosis, respectively (Deeks and Altman, 2004). In our study, the pooled PLRs and NLRs for circulating cfDNA assays were 11.82 (95% CI: 6.74–20.74) and 0.57 (95% CI: 0.41–0.80), respectively. Compared to healthy controls, patients with echinococcosis were approximately 11.82 times more likely to have a positive cfDNA test, with an error rate of approximately 57% when a true-negative was determined in a negative cfDNA test. These results demonstrate high specificity, low sensitivity, high accuracy, and poor robustness. This may be due to the variable quality of the literature and the fact that testing techniques and experimental design concepts have improved in subsequent studies, or to the fact that some of the studies had lower test accuracy and fewer cases. In addition, the zero-value correction method may have a negative impact on small studies. In addition, a DOR of 1 indicates that the test is unable to differentiate between diseased and healthy individuals without the disease (Glas et al., 2003). The pooled DORs in our study was 36.63 (95% CI: 13.75–97.59), suggesting a high overall accuracy.

cfDNA is an extracellular double-stranded nuclear and mitochondrial DNA fragment that can be found in different body samples and tissues, such as blood, urine, saliva (Jiang and Lo, 2016; Snyder et al., 2016), stool (Diehl et al., 2008), sputum (van der Drift et al., 2008), and cerebrospinal (Wang et al., 2015), peritoneal (Pajek et al., 2010), synovial (Leon et al., 1981), lymph and amniotic fluids (Wu et al., 2014). However, they are more abundant in blood and urine samples. The guidelines by Meddeb et al. (2019) also suggest that plasma is a better source for cfDNA analysis than serum (Meddeb et al., 2019). The sample types included in the literature we included in this meta-analysis included plasma, serum, and urine. The subgroup analyses supported this finding, with three sensitivities of 0.87, 0.19, and 0.25, PLRs of 13.71, 8.93, and 9.29, NLRs of 0.17, 0.85, and 0.51, DORs of 149.03, 13.36, and 14.96, respectively, and AUC values of 0.97, 0.86, and 0.72, respectively, suggesting that plasma-based assays show a higher level of accuracy. Some studies have suggested that because the growth process of *Echinococcus multilocularis* is different from that of *Echinococcus granulosus* and because necrotic parasite tissue and actively proliferating tissue of *Echinococcus multilocularis* tend to be mixed together and lack a clear margin between the tissue and human tissue, its cysts can release more cfDNA into the circulation than those of *Echinococcus granulosus* (Hadipour et al., 2023). The statistical results we derived showed that the accuracy of cfDNA detection was higher in the AE group, compared to CE, with sensitivities of 0.57 versus 0.35, PLRs of 13.07 versus 9.47, NLRs of 0.54 versus 0.57, DORs of 62.12 versus 19.38, and AUC values of 0.95 versus 0.90, respectively. On the data based on the subgroups of country origin and year of publication, we can see that studies originating from China or articles published after 2020 have better accuracy than the corresponding groups, and we hypothesize that the reason for this may be the limited research on the biological characteristics of echinococcosis in the early days and the immature cfDNA extraction technology that caused some studies to overestimate the diagnostic efficacy of cfDNA in echinococcosis. This performance is also reflected in the later meta-regression analysis. Despite the small sample sizes of each included study, our subgroup analyses showed that studies with large sample sizes showed better accuracy than those with small sample sizes, although regression analyses were not suggestive of this as a source of heterogeneity.

The common characteristics of cfDNA have been reported to be small length, low richness, and rapid degradation. Under normal conditions, their concentration in 1 mL of human plasma is about 1–10 ng, but under certain circumstances or after exercise, their concentration increases to hundreds of nanograms (Lo et al., 1999; Glas et al., 2003; Kamat et al., 2010; Breitbach et al., 2014; Jiang and Lo, 2016; Volik et al., 2016). The size of cfDNA has been estimated to vary between about 40–200 base pairs (bp), with a main peak of about 166 bp (Snyder et al., 2016; Zhang et al., 2016; Mouliere et al., 2018). They have a half-life of about 10–15 min and are usually cleared by the liver (Zeerleder, 2006). Considering the large heterogeneity of the included literature in terms of sensitivity, we utilized the REML method based on the mada package of the R software to conduct regression analyses for sensitivity. Our findings revealed that the *p*-values for the five dimensions of “country of origin,” “sample type,” “year of publication,” “assay methods,” and “target sequence” were all <0.05, indicating that they may be contributing to the heterogeneity in sensitivity. This indirectly suggests that the diagnostic efficacy of cfDNA for *Echinococcus granulosus* has been enhanced due to advancements in assay methods and target sequence design since 2020.

Although the combined DOR in our study was 36.63 (95% CI:13.75–97.59), suggesting that the overall accuracy of cfDNA for the diagnosis of echinococcosis is high, there are still some limitations to our study. It is mainly reflected in the following aspects: first, there are some unavoidable limitations in literature search, for example, the scope of search is limited to the published research, so unpublished research such as conference papers cannot be obtained, which may lead to missing for some relevant gray literature. Additionally, the search language is limited to Chinese and English, which may result in missing related research published in other languages. These may lead us to retrieve less comprehensive literature. In terms of quality assessment, the use of blinded testing and blinded judgment minimizes diagnostic predisposition, whereas the majority of all our included studies did not report whether a blinded test was used, and therefore, may lead to biased results. Second, some studies and expert consensus state that preoperative pathologic examination of biopsy samples is not recommended due to the risk of proto-cephalic node transmission and allergic reactions during echinococcosis biopsies. A combination of imaging and laboratory techniques is recommended for a correct diagnosis (Brunetti and Junghanss, 2009; Hadipour et al., 2023). For this reason, some of the studies we included did not confirm the diagnosis pathologically, but instead diagnosed it using imaging or serologic techniques, and although these do not violate the diagnostic criteria for echinococcosis, they still do not completely exclude the possibility of causing bias. In addition, it is important to note that four of the seven articles we ultimately included were from western China, with fewer relevant studies from other countries, which may have created a sample selection bias, coupled with the fact that for some subgroup analyses, the number of available studies was relatively small, limiting the generalizability of such pooled accuracy estimates. Interestingly, we found that the target sequence may contribute to one of the sources of heterogeneity. Several studies have found that most of the *Echinococcus* cfDNA in the blood of echinococcosis patients was from the nucleus, not from the mitochondrion (Ji et al., 2020; Zhao et al., 2021), possibly the reason for the poor performance of mitochondrial DNA. In addition, if the target region was not released into the blood circulation, or if there was a mismatch of the target regions, then the sensitivity would be low too (Weerakoon and McManus, 2016), Because of the lower abundance of cfDNA, if the sequence targeting the nuclear SnRNA is not specific enough or cannot be matched, it will also result in poor detection performance, whereas on the contrary, the studies with better sensitivity, which use genomic repeat region sequences or high-throughput sequencing techniques to try to better target the target cfDNA sequences, have achieved better results. So further exploration of the *Echinococcus* cfDNA characteristics in the plasma of both CE and AE patients might resolve the controversy. The accuracy of the reference genomes of both *E. granulosus* and *E. multilocularis* is still open to question, and the results could be further improved when better reference genomes are published. Finally, it has been reported that no statistically significant differences were found between patients’ cfDNA levels and the stage of echinococcosis, metastasis, number of lesions, and shape of lesions (Fan et al., 2021). However, there are reports confirming the potential of plasma cfDNA to be used as a biomarker in the therapeutic monitoring of echinococcosis (Zhao et al., 2021). Several studies have shown that cfDNA can be used for diagnosis, treatment response, and prediction of prognosis in many diseases. To date, they have been widely and satisfactorily applied in clinical practice and medical research, such as prenatal testing (Norwitz and Levy, 2013), tumor detection (Heitzer et al., 2015), organ transplantation monitoring, and pathogen detection (Snyder et al., 2011; De Vlaminck et al., 2014; Schütz et al., 2017; Blauwkamp et al., 2019). However, the number of these studies on echinococcosis is still quite limited. Therefore, the results obtained in our meta-analysis need to be confirmed by further large-scale studies in different countries, ethnic backgrounds, sample sizes, specimen types, detection methods, etc.

## 5 Conclusion

In conclusion, existing evidence indicates that the combined specificity of circulating cfDNA for echinococcosis is high. However, the combined sensitivity performance is unsatisfactory due to significant inter-study heterogeneity. This study represents the first meta-analysis and systematic evaluation of the feasibility of cfDNA as a diagnostic tool for echinococcosis. To strengthen the validity and accuracy of our findings, further large-scale prospective studies are required. These studies should focus on validating the conclusions drawn from this analysis and exploring the potential applicability of cfDNA, either alone or in conjunction with traditional markers, as a diagnostic biomarker for echinococcosis. By providing additional usable material, these studies can help us better understand the factors that influence diagnostic accuracy and improve the reliability of our conclusions.

## Data availability statement

The original contributions presented in the study are included in the article/supplementary material, further inquiries can be directed to the corresponding author.

## Author contributions

XL: Conceptualization, Formal analysis, Methodology, Writing – original draft, Writing – review & editing. PJ: Formal analysis, Investigation, Methodology, Software, Writing – original draft, Writing – review & editing. JM: Methodology, Supervision, Validation, Writing – review & editing. ZL: Methodology, Supervision, Writing – review & editing. JZ: Methodology, Supervision, Writing – review & editing. XW: Data curation, Methodology, Writing – review & editing. JA: Methodology, Software, Writing – review & editing. JC: Investigation, Methodology, Writing – review & editing. YL: Data curation, Formal analysis, Writing – review & editing. PC: Data curation, Validation, Writing – review & editing. CC: Validation, Writing – review & editing. XA: Formal analysis, Investigation, Methodology, Supervision, Writing – review & editing.
